# How relationship‐quality with family, peers, and teachers relates to adolescents' bicultural identity integration (BII)

**DOI:** 10.1111/jora.70321

**Published:** 2026-11-13

**Authors:** Paula Duwe, Peter F. Titzmann

**Affiliations:** ^1^ Department of Psychology Leibniz University Hannover Hanover Germany

**Keywords:** bicultural identity integration (BII), biculturalism, ethnic minority, ethnic‐cultural identity, family relationships, peer relationships, teacher relationships

## Abstract

Family, peers, and teachers are important socialization agents that often represent different cultural orientations for ethnically minoritized adolescents. This study examined how positive relationships to these socializing agents contribute to Bicultural Identity Integration (BII) among 226 minoritized adolescents in Germany (*M*_age_ = 12.9, 51.1% female, diverse national backgrounds, 27.1% first‐ and 72.9% second‐generation immigrants). Multinomial logistic regression analyses tested whether positive family climate, peer acceptance, and positive teacher‐generated classroom climate—independently and in combination—predict membership in five previously identified BII trajectories [two time points, 2 years apart, 2019–2021, identified through latent class growth analysis] (*p* < .001, *R* ^2^ = .238). Findings revealed that, particularly, the combination of multiple high‐quality relationships had synergistic effects in predicting membership in the more resolved BII trajectory subgroups High Integrated BI (*n =* 49, 21.6%) and Conflict Resolution (*n =* 18, 7.9%), while lower relationship quality predicted membership in the less resolved, Ambivalent BII (*n* = 69, 30.5%) subgroup. Hence, sustained positive engagement with multiple socialization agents provides minoritized adolescents with relational scaffoldings necessary to navigate and harmonize multiple cultural identities.

The growth of migration movements and cultural diversity in contemporary societies is a trend that has been on the rise globally. According to the United Nations Population Division (2024), the number of international migrants reached nearly 304 million in 2024, and ongoing geopolitical crises, such as the war in Ukraine or the armed conflict in Sudan, continue to add to these numbers. As a result, a substantial and growing proportion of young people worldwide are growing up with multiple cultures, which necessitates their engagement in acculturation processes, that is, ongoing processes of adaptation and negotiation between the cultural norms, values, and practices of the minority (ethnic‐heritage) and of the majority (national) culture (Berry, 1997). Supporting ethnically minoritized youth in this regard is essential for promoting their positive adjustment and, hence, is a crucial responsibility of contemporary societies. Germany, one of the largest destination countries in Europe, counted around 16.8 million international migrants in 2024 (United Nations International Migrant Stock, 2024), and 42% of all German children under 15 are of immigrant origin (i.e., they or at least one of their parents were born in another country) (BIB, 2022). This growing demographic trend underscores the need to better understand and support the adaptation of ethnically minoritized youth and to ensure equitable opportunities for their future. Promoting their successful adjustment and integration is not only essential for their individual development and well‐being but also critical for long‐term societal cohesion in increasingly multicultural societies.

Navigating ethnic minority cultural and majority cultural identities into a coherent sense of self is an acculturative‐developmental task that, if successfully resolved, has the potential to foster minoritized adolescents' adjustment and well‐being (Suárez‐Orozco et al., 2018). Bicultural Identity Integration (BII) captures an individual's subjective experience of this task by reflecting how individuals cognitively organize and affectively experience their ethnic minority and majority cultural identities. It provides a promising framework for understanding interindividual variability in the way these cultural identities are integrated and reflects the extent to which they are perceived as compatible or conflicting (Benet‐Martínez et al., 2021). Previous studies have found that developing blended and harmonious bicultural identities (High Integrated) is consistently linked to more adaptive psychosocial outcomes among minoritized individuals, and may serve as a key resource for supporting psychological well‐being and adaptation (Benet‐Martínez et al., 2021; Cheng et al., 2014; Duwe et al., 2026; Schwartz et al., 2015). However, there is still limited understanding of how adolescents experience and navigate the intersection of their bicultural identities. Towards this aim, longitudinal research on adolescents in Germany has identified five distinct BII trajectory subgroups, highlighting substantial heterogeneity in BII experiences (Duwe et al., 2026). The factors driving this variability in adolescents' BII experiences, however, remain largely unexplored. Socialization and identity theories suggest that proximal relationships (i.e., family, peers, teachers) may play an important role in shaping adolescents' BII because they provide key contexts for (cultural) identity formation and can facilitate the acquisition of cultural knowledge, norms, values, and social skills that foster a sense of belonging within social and cultural groups (Bowlby, 1973; Branje et al., 2021; Grusec & Hastings, 2014; Titzmann et al., 2024; Vygotsky, 1978). However, the role of supportive relationships with family, peers, and teachers in shaping adolescents' BII remains largely unknown.

Therefore, the current study aims to extend knowledge regarding how proximal relationships contribute to the BII development of minoritized adolescents. Specifically, it examined how relationship quality with family, peers, and teachers (both independently and in combination) predicts membership in the five previously identified BII trajectory subgroups (Duwe et al., 2026).

## Bicultural identity integration (BII)

Bicultural Identity Integration (BII) originates from acculturation theory (Berry, 1997) and offers a construct for understanding interindividual differences in how individuals manage multiple cultural identities, typically their ethnic minority cultural identity and their national majority cultural identity (Benet‐Martínez et al., 2021). For ethnically minoritized adolescents, BII represents a central acculturation task (Suárez‐Orozco et al., 2018), and its successful resolution has been linked to positive psychosocial adaptation outcomes (Benet‐Martínez et al., 2021; Cheng et al., 2014; Duwe et al., 2026; Schwartz et al., 2015). BII focuses on whether individuals perceive their cultural identities as compatible or in conflict (Benet‐Martínez et al., 2021; Huynh et al., 2011), encompassing both the cognitive and affective aspects of how these identities intersect and are organized within the self (Benet‐Martínez et al., 2021; Huynh et al., 2018).

Four dimensions have been developed to describe interindividual differences in BII, comprising two cognitive and two affective aspects (Duwe & Titzmann, 2024). These dimensions are typically assessed using the Bicultural Identity Integration Scale (BIIS‐2), which builds on earlier BII measures (BIIS‐1) by providing a more comprehensive and psychometrically refined assessment of bicultural identity integration, based on a larger item pool and allowing for a more precise operationalization of these four dimensions (Duwe & Titzmann, 2024; Huynh et al., 2018). The cognitive aspect of BII is captured by two dimensions, Blendedness and Compartmentalization (Duwe & Titzmann, 2024; Huynh et al., 2018), drawing on theoretical perspectives of identity fusion and/or alternation (LaFromboise et al., 1993) and Social Identity Complexity (Roccas & Brewer, 2002). Blendedness reflects the perception that one's cultural identities are integrated and overlapping (Cheng et al., 2014; Meca et al., 2020), whereas Compartmentalization refers to cultural identities being perceived as distinct and separated, without efforts to reconcile them (Roccas & Brewer, 2002). The affective aspect of BII is represented by the two dimensions of Harmony and Conflict (Duwe & Titzmann, 2024). Harmony reflects the positive affective experience of having balanced and compatible cultural identities (Benet‐Martínez et al., 2021; Huynh et al., 2018). In contrast, Conflict captures the experience of tension or incompatibility, where individuals feel torn between the expectations or practices associated with their multiple cultural identities (Benet‐Martínez et al., 2021; Huynh et al., 2018). Knowledge about BII development among minoritized youth in European contexts is scarce. Existing findings from the United States may not generalize to these contexts, as they primarily relied on adult samples and earlier versions of the BII scale (BIIS‐1).

Moreover, recent research utilized a person‐oriented approach and identified subgroups of multicultural youth in Germany that differ in how they combine the four BII dimensions over time (Duwe et al., 2026) (Figure 1). These findings suggest substantial heterogeneity in BII trajectories and combinations not captured by variable approaches alone. Specifically, five distinct BII trajectory subgroups emerged (see Figure 1), differing in their association with psychological well‐being. These subgroups reflect previously established, but also novel BII configurations that vary in their levels and rates of change across BII dimensions. Two subgroups reflected more resolved forms of BII: The High Integrated BI trajectory subgroup, characterized by relatively stable high levels of Blendedness and Harmony and low levels of Compartmentalization and Conflict, and the Conflict Resolution trajectory subgroup, characterized by initially high levels across all four BII dimensions followed by significant change over time—most notably a pronounced decrease in BII Conflict (Duwe et al., 2026). Adolescents in both of these subgroups reported relatively high levels of psychological well‐being at T2. A third BII trajectory subgroup, Ambivalent BII, reflected a more unresolved state of BII characterized by medium to high and relatively stable levels across all four BII dimensions—Harmony, Conflict, Blendedness, and Compartmentalization—accompanied by a significant increase in Conflict over time. This subgroup showed an ambivalent BII pattern, with no clear orientation towards Blendedness or Compartmentalization and, simultaneously, mixed feelings of Harmony and Conflict. Adolescents in the Ambivalent BII subgroup reported the lowest levels of psychological well‐being at T2 compared to all other BII subgroups (Duwe et al., 2026).

**FIGURE 1:**
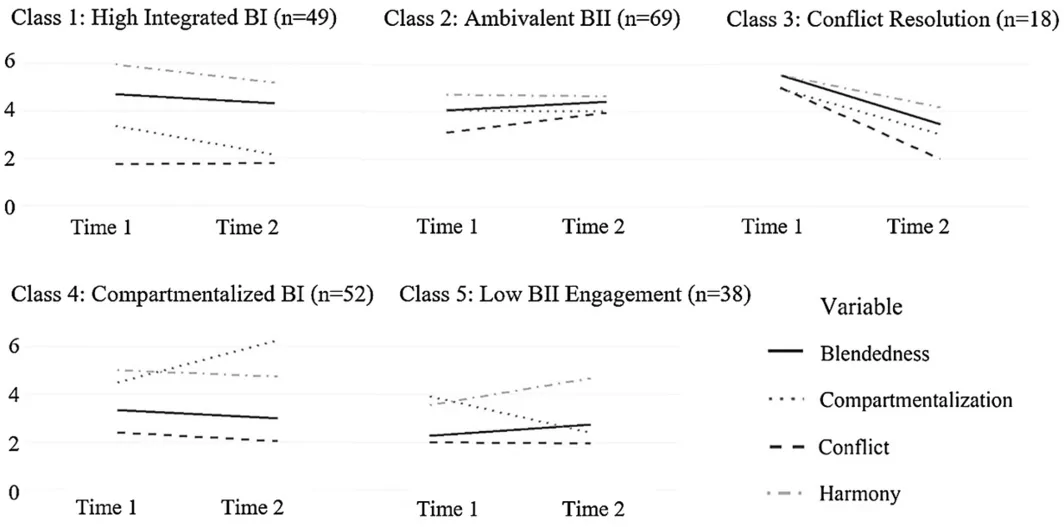
BII trajectory subgroups (Duwe et al., 2026). The term trajectories of BII dimensions across latent classes refers to average changes within each class between T1 and T2, because the study comprised two measurement points.

The remaining two BII trajectory subgroups (Compartmentalized BI and Low BII Engagement) were not classified as resolved or unresolved. The Compartmentalized BI subgroup showed high and increasing levels of Compartmentalization alongside high, stable Harmony, while Blendedness and Conflict remained low. The Low BII Engagement subgroup showed consistently low levels of Compartmentalization and Blendedness, indicating minimal engagement in cognitive organization of bicultural identities combined with low Conflict and moderate, increasing Harmony. Both of these BII trajectory subgroups reported medium‐high levels of psychological well‐being (Duwe et al., 2026). Hence, individuals in these two subgroups seem to experience that their cultural identities do not necessarily need to be combined to achieve satisfactory adaptation. These subgroups may be a particular result of the German context, where societal expectations for assimilation remain prevalent and where hyphenated identities are still not the norm, so that adolescents may seek ways to separate their identities (Duwe & Titzmann, 2024; Zick & Krott, 2021).

### BII and proximal relationships

The five longitudinal BII trajectory subgroups previously identified (Duwe et al., 2026) reflect different ways in which ethnically minoritized adolescents in Germany experience and navigate their bicultural identities, as well as how these trajectories are associated with their well‐being. It is an open question, however, why adolescents end up in these BII trajectory subgroups. Theoretically, identity and socialization theories may help fill this gap. *Identity theory* states that proximal relationships are crucial for identity development of all youth (both of immigrant and non‐immigrant origin). Social relationships provide important contexts in which individuals receive feedback about identity‐related roles, values, and ways of expressing themselves and, when relationship quality in these contexts is high, provide a secure foundation for identity exploration, which is helpful for strengthening or adjusting identity commitments (Bowlby, 1973; Branje et al., 2021). *Socialization theories* highlight the central role of culture and social interaction in cognitive development and socialization (Vygotsky, 1978). From this perspective, learning occurs through interactions with more knowledgeable others within individuals' proximal contexts. Hence, both identity and socialization theories argue in the same direction and endorse the role of proximal relationships in identity processes. In particular, family, peers, and teachers transmit relevant knowledge, norms, values, and social skills that enable individuals to participate effectively and develop a sense of belonging in a given society or social and cultural group (Grusec & Hastings, 2014). For ethnically minoritized adolescents, however, these proximal relationships often represent distinct cultural affiliations.

Families constitute primary socialization agents and are often the social context most closely affiliated with adolescents' ethnic heritage culture. Parents engage in multiple strategies of cultural transmission, commonly conceptualized as ethnic–racial socialization practices. These strategies include cultural socialization (i.e., the transmission of cultural values, customs, traditions, history, and pride), preparation for bias (i.e., preparing youth to cope with experiences of discrimination and racism), and egalitarian messages and attitudes, which emphasize equality across ethnic–racial groups (Hughes et al., 2006; Umaña‐Taylor & Hill, 2020). Empirical research links parents' engagement in cultural socialization to positive youth outcomes, including stronger ethnic minority identity and greater identity resolution (Hughes et al., 2006; Huguley et al., 2019). The influence of family socialization processes on adolescents' identity development, however, has predominantly been examined separately for ethnic minority and majority cultural identity, leaving limited knowledge about their role in how adolescents integrate multiple cultural identities. Although it can be assumed that a supportive family climate may result in stronger transmission of ethnic heritage values and identity (Miklikowska, 2016; Zagrean et al., 2022), high‐quality parent–child relationships can also provide a secure space for identity exploration (Crocetti et al., 2023), which should support adolescents' bicultural identity integration (BII). Among Hispanic adolescents in the U.S., for instance, more favorable family relationships were associated with integrated bicultural identities (characterized by high Blendedness and Harmony) (Schwartz et al., 2015).

Peers are becoming a major socialization agent during adolescence and are characterized by increasingly diverse cultural and ethnic backgrounds in most contemporary societies (Duwe & Titzmann, 2024; Meissner & Vertovec, 2016). Hence, in multicultural contexts, such as Germany, peers do not represent a specific (heritage or receiving) culture; rather, they represent multiple heritages and cultural patterns. As adolescents transition to secondary schools, interact with many diverse age‐mates, and simultaneously seek greater autonomy from parents, they cultivate positive peer relationships of various kinds (Titzmann et al., 2024). These relationships shape adolescents' social support systems as well as their normative, social, emotional, and identity development (Klimstra et al., 2013; Nawaz, 2011; Rassart et al., 2012). Being ethnically minoritized and experiencing acceptance and positive relationships within multicultural peer networks can support adolescents' bicultural identity integration (BII) in various ways. First, positive multicultural peer relations can increase perceptions of multicultural normativity, cultural group similarity, and permeability (Benet‐Martínez et al., 2021; Gaertner et al., 1993; Paluck, 2011), which supports adolescents' perceptions of compatibility of minority and majority cultures. Second, multicultural peers can serve as additional role models beyond those offered by parents (Bandura, 1986), providing diverse and complementary examples of possible ways to navigate multiple cultural identities. Together, these processes may help resolve ambivalent or conflicted BII and promote more integrated bicultural identities (Benet‐Martínez et al., 2021). However, empirical research on peer relationships and BII remains limited.

Teachers in multicultural schools are secondary socialization agents and play a crucial role in adolescents' social, emotional, and academic development (Titzmann et al., 2024). In Germany, the vast majority of teachers represent the social majority and are trained in majority‐culture institutions (Paizan et al., 2024). Hence, schools and teachers often transmit knowledge, values, norms, and acculturative expectations primarily aligned with the national majority culture, which often differs from those emphasized in the family context (Grundmann, 2021; Ward & Szabó, 2023). A positive teacher‐generated classroom climate benefits both minoritized and majority students by fostering high‐quality student‐teacher relationships, greater teacher‐student instrumental support, and more positive academic and psychological adjustment outcomes (Engels et al., 2021; Ialuna et al., 2024; Paizan et al., 2024; Roorda et al., 2017). Empirical evidence on how the teacher‐generated classroom climate relates to students' bicultural identity integration (BII) remains, however, inconclusive. From a theoretical perspective, a positive teacher‐generated classroom climate may promote BII in two ways. First, teachers and schools are primarily affiliated with the national majority culture and expose adolescents to its knowledge, norms, and practices. In doing so, they support students' cultural learning and transmission of majority values while providing opportunities to foster the development of a majority identity alongside ethnic minority identities. For example, they can incorporate culturally responsive teaching practices such as intercultural learning into the curriculum by combining teaching about national cultural norms, history, and civic values with more diverse content, examples, and materials that reflect students' ethnic‐cultural backgrounds and incorporate multicultural perspectives (Aral et al., 2024; Gay, 2018; Juang et al., 2024). Second, a positive teacher‐generated classroom climate in multicultural classes may signal norms of inclusivity that reflect majority‐group support for diversity, thereby enhancing perceived normativity of dual ethnic‐cultural identification and belonging (Schachner et al., 2016). Together, a positive teacher‐generated classroom climate in contemporary multicultural classrooms may facilitate ethnically minoritized students' BII.

### BII and interactions between proximal relationships

Following these arguments, each relationship (family, peers, teachers) may support adolescents' BII. Youth development is, however, shaped by dynamic interactions between individuals and multiple social relations. According to ecological developmental models, development emerges from complex, interdependent links between proximal microsystems (e.g., family, peers, teachers) and more distal contexts such as societal norms and institutions (Bronfenbrenner, 1981; Reich & Vandell, 2011; Suárez‐Orozco et al., 2018). Direct and indirect interactions across these social contexts can exert additive and synergistic effects beyond those of single microsystems, so that effects of one setting may depend on the presence of protective or risk factors in other contexts (Bronfenbrenner, 1981; Miklikowska & Bohman, 2020).

In terms of BII development, cross‐context dynamics between family, peers, and teachers are particularly relevant because each relationship tends to be differently affiliated with cultural norms and orientations. Positive experiences across these relationships therefore provide adolescents with safe spaces, secure foundations, and a broader range of identity‐relevant knowledge and feedback on how to acquire, negotiate, and reconcile multiple cultural frameworks (Branje et al., 2021; Crocetti et al., 2023; Suárez‐Orozco et al., 2018). Such relational resources are crucial for supporting the development and integration of multiple cultural identities. Accordingly, simultaneously maintaining positive relationships with family, peers, and teachers (affiliated with different cultural orientations) should be especially effective in reducing Ambivalent BII and fostering more resolved and integrated bicultural identity development (i.e., High Integrated BI, Conflict Resolution) during adolescence.

## Current study

Guided by theoretical perspectives on identity development (Branje et al., 2021), socialization (Meca et al., 2020; Titzmann et al., 2024; Vygotsky, 1978), and ecological development (Bronfenbrenner, 1981; Galliher et al., 2017; Reich & Vandell, 2011; Suárez‐Orozco et al., 2018), the present study examines how ethnically minoritized adolescents' relational experiences with family, peers, and teachers are associated with their trajectories of bicultural identity integration (BII). The BII trajectory subgroups have been previously identified and descriptively examined in earlier work (Duwe et al., 2026; see Figure 1) and are used in this study as the dependent variable. Building on these findings, the present study investigates how proximal relationships are associated with these distinct BII trajectory subgroups.

Positive relationships with family, peers, and teachers should facilitate cultural learning and transmission, and provide emotionally supportive and safe contexts that support adolescents' identity formation. Therefore, we expect that positive relationships with family, peers, and teachers (main effects) should be positively associated with membership in more resolved BII trajectory subgroups (High Integrated BI and Conflict Resolution), while lacking positive relationships with family, peers, and teachers (main effects) should be associated with membership in the less resolved BII trajectory subgroup (Ambivalent BII) *(H1a family, H1b peers, H1c teacher)*.

In addition, for minoritized adolescents, maintaining several positive relationships that likely comprise different cultural affiliations is expected to be especially beneficial for resolving bicultural identity integration (BII). To capture this interplay, we test three interaction terms (family × peer, family × teacher, and peer × teacher) and examine whether different combinations of relationship quality are associated with distinct BII trajectory subgroups. We hypothesize that adolescents who report multiple positive relationships should be more likely to follow more resolved BII trajectory subgroups (High Integrated BI, Conflict Resolution), compared to individuals with less resolved BII trajectories (Ambivalent BII) *(H2a family‐peer, H2b family‐teacher, H2c peer‐teacher)*.

Relatedly, a three‐way interaction among relationship qualities (family × peer × teacher) will be tested. However, given the higher‐order nature of three‐way interactions, their dependence on all lower‐order effects, and the greater statistical power required for their reliable detection, we do not formulate a specific hypothesis regarding this effect. Instead, the three‐way family × peer × teacher interaction will be examined exploratorily across all BII trajectory subgroups, and findings pertaining to this interaction will be interpreted with caution.

Moreover, we do not formulate specific hypotheses for the Compartmentalized BI and Low BII Engagement trajectory subgroups, as it is an open question whether these subgroups represent more or less resolved forms of BII. Accordingly, associations between relationship qualities (independently and in combination) and membership in these BII trajectory subgroups will be examined exploratorily. This approach may yield initial insights into the relational processes that could shape these BII subgroups and into the extent to which these trajectory subgroups may reflect more or less adaptive forms of BII.

## METHOD

### Participants and procedure

The present study draws on data from the project *Teacher–Parent Interactions: Improving the Academic Achievement of Ethnic Minority and Majority Adolescents* (Institut für Psychologie, 2024), funded by the Ministry for Science and Culture of Lower Saxony, Germany. Ethical approval from the Ethics Committee of Leibniz University Hannover was granted. Data were gathered at two time points between 2019 (T1) and 2021 (T2) in seven secondary schools in Hanover and its surrounding areas. Hanover is characterized by substantial cultural diversity, with 41.4% of residents reporting immigrant origin and 52% of minors holding dual citizenship (Landeshauptstadt Hannover, 2022). Prior to data collection, permission was secured from relevant school authorities, principals, and parents or legal guardians. Students completed paper‐and‐pencil questionnaires during regular class time under the supervision of teachers and trained research staff. Participants received a €10 voucher as compensation.

The sample of the present study comprised students who reported immigrant origin and provided data at both time points (*N* = 226). Participants who were missing at the second wave (*n* = 317) were compared to those retained in the study to examine potential attrition bias. These analyses indicated no statistically significant differences between groups in terms of BII dimensions, gender, or immigrant generation. However, students who did not participate at T2 were on average older at T1. Given the extended interval between assessments—partly due to delays related to the COVID‐19 pandemic—this age difference likely reflects school transitions, as some older adolescents had graduated by the time of the second data collection. Overall, attrition appears largely random. Immigrant origin was assessed based on students' and parents' countries of birth, distinguishing between first‐ and second‐generation immigrants. For first‐generation adolescents, length of residence in Germany was additionally recorded. On average, they had lived in Germany for 5.3 years (*SD* = 3.2), indicating that many were likely experiencing typical first‐generation acculturation processes (Titzmann et al., 2024). Across generations, students reported high levels of German language proficiency (scale range: 1–4; *M* = 3.49). The final sample consisted of 226 adolescents, of whom 27.1% were first‐generation and 72.9% second‐generation immigrants. The sample reflected diverse national backgrounds (45 different countries; see Supporting Information Material S1–S6 for an extensive overview). Countries were grouped into broad geographic regions for descriptive purposes, with MENA (Middle East and North Africa) used as a broad regional designation (Lelieveld et al., 2016). For countries outside MENA, geographic classifications followed the United Nations M49 regional recommendations (United Nations Statistics Division, 1999). 36.28% of mothers were born in countries of the MENA region (i.e., Türkiye, Syria, Lebanon), 13.27% in countries of Southern Europe (i.e., Serbia, Romania, Greece), 12.39% in countries of Eastern Europe (i.e., Russia, Ukraine, Poland), 11.06% in countries of Western Europe (i.e., France, Germany with fathers born abroad), 10.18% in countries of Sub‐Saharan Africa (i.e., Ghana, Nigeria, Senegal), 6.64% in countries of Southern Asia (i.e., Afghanistan, Pakistan, India), and 11.51% in other countries (i.e., countries in Central Asia, North America, South America). At T1, participants were on average 12.9 years old (*SD* = 1.19; range = 11–16), and 45.5% were female.

### Measures

All study materials were in German, and all scales were scored on a Likert scale ranging from 1 (strongly disagree or very poor) to 7 (strongly agree or very good).

#### BII trajectory subgroups

Bicultural Identity Integration was assessed using the BIIS‐2 (Huynh et al., 2018), which originally comprised two bidirectional dimensions (Harmony vs. Conflict and Blendedness vs. Compartmentalization) and 17 items. In the present study, the adapted German version for ethnically minoritized adolescents was used, which includes four distinct subscales (Harmony, Conflict, Blendedness, Compartmentalization) and 16 items (see Duwe & Titzmann, 2024, for a detailed report on this scale). Based on this adapted four‐dimensional measure, five BII trajectory subgroups were identified in a previous study using Latent Class Growth Analysis (LCGA) (Duwe et al., 2026), and are used as the dependent variable in the current study. As described in the introduction of this study, the five trajectory subgroups comprise the *High Integrated BI* subgroup (overall high levels of Harmony and Blendedness, with slight decrease in Harmony from T1 to T2, rather low levels of Conflict and Compartmentalization), the *Ambivalent BII* subgroup (medium‐high levels in all four BII dimensions, significant increase in Conflict from T1 to T2), the *Conflict Resolution* subgroup (high levels in all four BII dimensions at T1 and a significant decrease of all dimensions from T1 to T2, with the greatest decrease in Conflict), the *Compartmentalized BI* subgroup (overall high and significantly increasing levels of Compartmentalization, combined with high and stable levels of Harmony, while Blendedness and Conflict remained comparatively low), and the *Low BII Engagement* subgroup (low levels of Compartmentalization, Blendedness, and Conflict and medium levels of Harmony that significantly increased from T1 to T2) (see subgroup description in the introduction, Figure 1, Duwe et al., 2026).

#### Relationship qualities

The Family Climate scale is an eight‐item scale developed by Schneewind and Weiß (1996) to assess family relations in the German context and was assessed at T1. The original version consisted of two subdimensions: communication (*We can calmly discuss problems with each other*) and cohesion (*In our family, we feel very close to one another*). CFA, however, indicated a good psychometric fit for the one‐factor solution (CFI = .922; RMSEA = .097; SRMR = .050) (a second factor did not improve the model fit), and both dimensions were highly correlated (*r* = .90, *p* = .000). Therefore, a single overall Family Climate score was used in subsequent analyses. The scale demonstrated excellent internal consistency (*α* = .91; *ω* = .91).

Peer Acceptance was assessed using three items adapted from the Loneliness and Social Dissatisfaction Scale by Asher et al. (1984) and was assessed at T1. The items that capture positive peer relations (*I have many friends in my class, I am very popular with the other children in my class, I get along well with my classmates*) were selected. The adapted scale demonstrated good internal consistency (*α* = .82; *ω* = .82). Because the scale comprised only three items, the CFA model was just‐identified, and the model fit was perfect by definition.

The Teacher‐Generated Classroom Climate was assessed with a scale developed by Meyer et al. (2007) to assess German students' perceptions of their teacher's behavior with regard to relational and learning‐supportive classroom factors, such as fairness and respectful treatment of all students (*There are students in my class who are treated preferentially by my teacher* (reverse‐coded); *My teacher lets me finish speaking when it is my turn*). The scale was assessed at T1 and comprised eight items with acceptable internal consistency (*α* = .75; *ω* = .72). Results of confirmatory factor analysis (CFA), including a method factor for negative items, indicated good model fit (CFI = .961; RMSEA = .065; SRMR = .067).

Demographic variables included age, gender, place of birth, place of birth of the parents, and self‐reported German language proficiency and served as sample description as well as control variables for sensitivity analyses. Age was assessed as an open‐ended question asking participants to report their age in years. Responses were coded into a continuous age variable ranging from 11 to 16 years at T1. Gender was assessed as a categorical variable (1 = female, 2 = male, 3 = diverse). Participants also reported their own country of birth and the countries of birth of both parents in open‐ended response fields. Based on these responses, an immigrant generation variable was derived, with adolescents born outside Germany classified as first‐generation immigrants and adolescents born in Germany with at least one parent born abroad classified as second‐generation immigrants. German language proficiency was assessed using four self‐reported items measuring adolescents' perceived ability to understand, read, speak, and write in German *(How well can you understand German?)*. Responses were scored on a Likert scale ranging from 1 (not at all) to 4 (very good) (4 items; *α* = .85; Titzmann & Gniewosz, 2018).

### Statistical analysis

First, all relationship quality variables were mean‐centered, and interaction terms were computed in SPSS (Version 29). Multicollinearity was assessed by examining correlation coefficients and variance inflation factors (VIFs) in a linear regression model including all mean‐centered predictors (main effects and interaction terms). Significant correlation coefficients ranged from 0.186 to 0.453, and VIF values ranged from 1.10 to 1.30, indicating low concerns for multicollinearity (VIF < 10; Bowerman & O'Connell, 1990). Subsequently, a multinomial logistic regression analysis was conducted, with mean‐centered relationship quality variables (main effects) and their interaction terms (family climate × peer acceptance, family climate × teacher‐generated classroom climate, peer acceptance × teacher‐generated classroom climate) entered as predictors and BII trajectory subgroup membership entered as the categorical outcome variable (Agresti, 2002; Aiken et al., 2010). The High Integrated BI subgroup was selected as the primary reference group because it represents a more resolved form of BII and has previously been associated with the highest levels of psychological adaptation (Duwe et al., 2026; Schwartz et al., 2015). In subsequent analyses, we also used the Ambivalent BII subgroup as a second reference group because this subgroup reflects a rather unresolved state of BII that has previously been associated with the lowest levels of psychological adaptation (Duwe et al., 2026). Together, these complementary reference groups enable comparisons relative to both the most and least adaptive forms of BII. This analytic strategy was used to examine associations between relationship quality in adolescents' proximal relational contexts and BII trajectory subgroup membership.

## RESULTS

### Preliminary analyses

Overall, the predictor variables showed weak to moderate positive associations. Peer acceptance was positively correlated with family climate (*r* = .331, *p* = .001) and teacher‐generated classroom climate (*r* = .191, *p* = .001), and family climate was positively correlated with teacher‐generated classroom climate (*r* = .259, *p* = .001). Descriptive statistics for all predictor variables by BII trajectory subgroup membership are reported in Table 1.

**TABLE 1:** Descriptive statistics of predictor variables by BII subgroup membership.

	Family climate		Peer acceptance		Teacher‐generated classroom‐climate	
	*M*	SD	*M*	SD	*M*	SD
BII subgroup						
High integrated BI	5.88^a^	1.19	5.43^ab^	1.38	5.58^a^	0.98
Conflict resolution	5.77^a^	1.25	6.04^b^	1.13	5.28^ab^	1.01
Ambivalent BII	5.64^a^	1.25	5.19^a^	1.42	4.88^b^	1.03
Compartmentalized BI	6.03^a^	0.90	5.03^a^	1.47	5.21^ab^	1.10
Low BII engagement	5.84^a^	1.17	5.53^ab^	1.19	5.35^a^	0.95

*Note*: Means sharing a common superscript are not significantly different at *p* < .05.

### Prediction of BII trajectory subgroups

To examine how relationship quality with family, peers, and teachers is associated with BII trajectory subgroups, we employed multinomial logistic regression analyses. The overall model was significant, *χ* ^2^(24) = 52.397, *p* < .001, and explained 23.8% of the variance (Cox and Snell pseudo‐r‐squared .238), indicating significant associations of predictor variables with the likelihood of BII subgroup membership. When examining effects across BII subgroups (Table 2, Table 3), higher levels of family climate did not increase the likelihood of membership in more resolved BII subgroups (i.e., High Integrated BI or Conflict Resolution) (*H1a not supported*). Higher peer acceptance increased the likelihood of belonging to the Conflict Resolution subgroup, relative to the likelihood of being a member of the Ambivalent BII subgroup (*OR* = 2.33, *p* = .033) (*supporting H1b*). Higher teacher‐generated classroom climate increased the likelihood of belonging to the High Integrated BI subgroup relative to the likelihood of membership in the Ambivalent BII subgroup (*OR* = 2.13, *p* = .002) (*supporting H1c*).

**TABLE 2:** Multinomial logistic regression with predictor variables as Ivs, BII subgroup membership as DV, and high integrated BI as reference group.

	Ambivalent BII				Conflict resolution				Compartmentalized BI				Low BII engagement			
	*B*	Wald	OR (95% CI)	*p*	*B*	Wald	OR (95% CI)	*p*	*B*	Wald	OR (95% CI)	*p*	*B*	Wald	OR (95% CI)	*p*
RG: high integrated BI																
Family climate	−.02	.00	.98 (.64–1.49)	.930	−.39	1.37	.67 (.34–1.30)	.241	.55	4.46	1.75 (1.04–2.94)	.035	−.15	.40	.85 (.52–1.39)	.527
Peer acceptance	−.06	.10	.94 (.66–1.33)	.745	.79	3.83	2.20 (.99–4.87)	.050	−.31	2.85	.73 (.50–1.05)	.091	.41	2.68	1.50 (.92–2.46)	.101
Teacher classroom climate	−.76	9.83	.46 (.29–.75)	.002	−.43	1.17	.64 (.29–1.42)	.278	−.43	1.67	.72 (.45–1.17)	.196	.03	.01	1.03 (.57–1.83)	.915
Interaction family × peer	−.15	.91	.85 (.62–1.17)	.339	.35	2.32	1.42 (.90–2.25)	.127	.22	1.84	1.25 (.90–1.73)	.174	.11	.44	1.12 (.79–1.59)	.507
Interaction family × teacher	.16	.62	1.17 (.79–1.73)	.428	−.13	.20	.87 (.49–1.54)	.651	−.31	1.67	.72 (.45–1.17)	.195	.41	3.21	1.51 (.96–2.40)	.073
Interaction peer × teacher	−.36	4.25	.69 (.49–.98)	.039	−.13	.18	.87 (.46–1.63)	.667	−.02	.02	.97 (.97–.69)	.875	−.72	8.29	.48 (.29–.79)	.004

Abbreviations: OR, odds ratio; RG, reference group.

**TABLE 3:** Multinomial logistic regression with predictor variables as Ivs, BII subgroup membership as DV, Ambivalent BII as reference group.

	High integrated BI				Conflict resolution				Compartmentalized BI				Low BII engagement			
	*B*	Wald	OR (95% CI)	*p*	*B*	Wald	OR (95% CI)	*p*	*B*	Wald	OR (95% CI)	*p*	*B*	Wald	OR (95% CI)	*p*
RG: Ambivalent BII																
Family climate	.02	.00	1.01 (.67–1.55)	.930	−.37	1.30	.68 (.35–1.31)	.254	.57	5.03	1.78 (1.07–2.95)	.025	−.14	.36	.86 (.55–1.37)	.545
Peer acceptance	.06	.10	1.05 (.74–1.49)	.745	.84	4.54	2.33 (1.07–5.10)	.033	−.26	2.32	.77 (.55–1.07)	.127	.46	4.00	1.59 (1.01–2.52)	.045
Teacher classroom climate	.76	9.83	2.13 (1.33–3.43)	.002	.32	.68	1.38 (.64–2.96)	.405	.44	3.72	1.55 (.99–2.43)	.054	.79	8.10	2.20 (1.28–3.80)	.004
Interaction family × peer	.15	.91	1.16 (.85–1.59)	.229	.50	5.03	1.66 (1.06–2.59)	.025	.37	5.69	1.45 (1.07–1.99)	.017	.27	2.68	1.31 (.94–1.81)	.102
Interaction family × teacher	−.16	.62	.85 (.57–1.26)	.428	−.28	1.05	.74 (.43–1.29)	.304	−.47	4.03	.62 (.39–.98)	.045	.25	1.71	1.29 (.87–1.91)	.190
Interaction peer × teacher	.36	4.25	1.43 (1.01–2.02)	.039	.22	.46	1.25 (.65–2.36)	.494	.33	3.94	1.39 (1.00–1.94)	.047	−.36	2.26	.69 (.43–1.11)	.133

Abbreviations: OR, odds ratio, RG, reference group.

Examination of interaction effects indicated that the co‐occurrence of positive relationships with family, peers, and teachers amplified some of the observed effects. Specifically, high levels of family climate and peer acceptance increased the likelihood of membership in the Conflict Resolution subgroup, relative to membership in the Ambivalent BII subgroup (*OR* = 1.66, *p* = .025) (*supporting H2a*) (Figure 2). In addition, simultaneous positive teacher‐generated classroom climate and high peer acceptance significantly increased the likelihood of membership in the High integrated BI subgroup, relative to membership in the Ambivalent BII subgroup (*OR* = 1.43, *p* = .039) (*supporting H2c*) (Figure 3). Unexpectedly, the co‐occurrence of a positive family climate and a positive teacher‐generated classroom climate did not significantly increase the likelihood of belonging to more resolved BII subgroups (*H2b not supported*).

**FIGURE 2:**
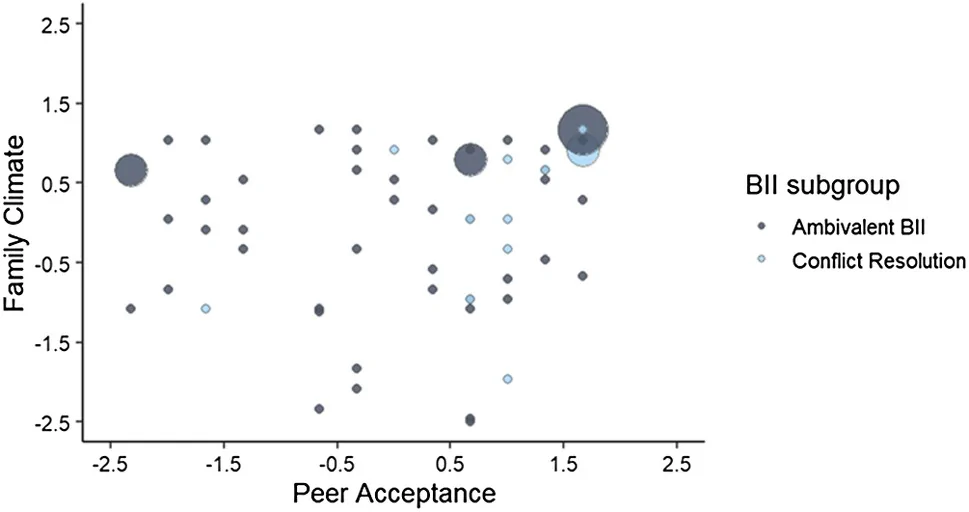
Peer acceptance and family climate (mean‐centered) by BII subgroup.

**FIGURE 3:**
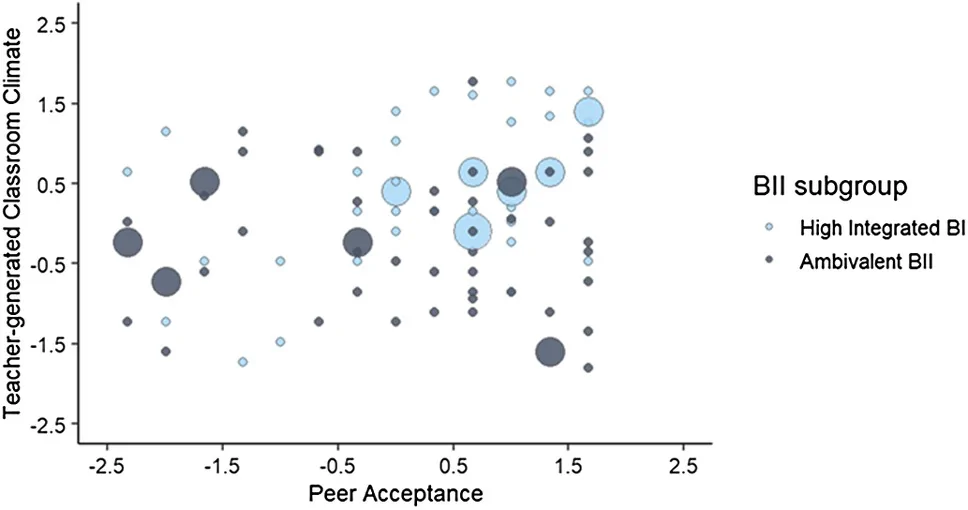
Teacher‐generated classroom climate and peer acceptance (mean‐centered) by BII subgroup.

Moreover, the two additional BII trajectory subgroups that are not clearly defined as resolved or unresolved also showed significant associations with relationship quality indicators. Higher levels of family climate were associated with membership in the Compartmentalized BI subgroup compared to both the High Integrated BI (*OR* = 1.75, *p* = .035) and the Ambivalent BII (*OR* = 1.78, *p* = .025) subgroups. In addition, a positive interaction between family climate and peer acceptance (*OR* = 1.45, *p* = .017) as well as between peer acceptance and teacher‐generated classroom climate (*OR* = 1.39, *p* = .047) increased the likelihood of membership in the Compartmentalized BI subgroup, relative to the Ambivalent BII subgroup. Moreover, the interaction between family climate and teacher‐generated classroom climate significantly differentiated the Compartmentalized BI and Ambivalent BII subgroups (*OR* = 0.62, *p* = .047). To facilitate the interpretation of the interaction effects, mean‐centered relationship variables were descriptively examined across BII trajectory subgroups (Supporting Information Material S1–S6). Descriptive statistics indicated that the positive interaction effect found for the Ambivalent BII subgroup was characterized by simultaneously low levels (below 0) of both family climate and teacher‐generated classroom climate, whereas the Compartmentalized BI subgroup showed relatively higher family climate despite less supportive teacher relationships. Plots illustrating the distribution of relationship qualities associated with significant interaction effects between the Compartmentalized BI and Ambivalent BII subgroups are provided in the Supporting Information Material S1–S6. Lastly, the likelihood of belonging to the Low BII Engagement subgroup was associated with higher levels of teacher‐generated classroom climate (*OR* = 2.20, *p* = .004) and peer acceptance (*OR* = 1.59, *p* = .045) (main effects), compared to the Ambivalent BII subgroup.

### Sensitivity analysis

To ensure that results were independent of demographic characteristics, we conducted sensitivity analyses to examine the robustness of our findings (see Supporting Information Material S1–S6). Although these analyses were underpowered given the number of estimated effects and the sample size in BII trajectory subgroups, the pattern of findings remained largely unchanged, supporting the robustness of the main results. One interaction effect no longer reached conventional levels of statistical significance, but the corresponding regression coefficient and odds ratios remained comparable to those obtained in the primary analyses. Overall, this slight attenuation of effects is likely due to a reduction in statistical precision rather than a substantive change due to additional control variables.

### Exploratory analysis: Three‐way interaction of family, peers, and teachers

Exploratory analyses were conducted by adding a *three‐way interaction* term (family climate × peer acceptance × teacher‐generated classroom climate) to the regression model to examine whether high‐quality relationships across all three socialization agents jointly predicted BII trajectory subgroup membership and explained additional variance. These analyses should be interpreted cautiously, as some BII trajectory subgroups were relatively small, limiting statistical power to detect higher‐order interaction effects. Nevertheless, with the inclusion of the three‐way interaction, the main effects and two‐way interaction effects were largely replicated (see Supporting Information Material S1–S6). In addition, the three‐way interaction yielded one significant result: The simultaneous experience of positive relationships with family, peers, and teacher decreased the likelihood of belonging to the Conflict Resolution subgroup, compared to membership in the High Integrated BI subgroup (*OR* = 0.64, *p* = .043).

## DISCUSSION

The present study aimed to extend existing knowledge on how proximal relationships contribute to the development of bicultural identity integration (BII) during adolescence. Our findings show that positive relationships with family, peers, and teachers both independently and in combination support more resolved and adaptive forms of BII. In this sense, high‐quality relationships with family, peers, and teachers function as resources for BII, probably because they provide multiple and complementary spaces in which adolescents explore, negotiate, and receive feedback on how to manage and reconcile multiple (culturally oriented) environments and identities (Bowlby, 1973; Branje et al., 2021; Crocetti et al., 2023; Suárez‐Orozco et al., 2018). It is important to note that mean levels of relationship quality were generally high across all BII trajectory subgroups, indicating an overall well‐functioning relational environment in our sample. Nevertheless, distinct constellations of relationship quality with family, peers, and teachers were associated with membership in different BII trajectory subgroups. Hence, each relationship may contribute unique aspects to the processes of negotiating and integrating multiple cultural identities.

### Socialization agents and BII

The *family* functions as a primary socialization agent and is typically adolescents' first and most influential source of ethnic heritage transmission (Hughes et al., 2006; Umaña‐Taylor & Hill, 2020). Beyond fostering heritage cultural knowledge and identity, warm and supportive family relationships may also provide a secure base from which adolescents can explore and negotiate the interplay of their heritage and national cultural identities (Crocetti et al., 2023; Miklikowska, 2016; Zagrean et al., 2022), which in turn should increase the likelihood of developing more resolved and integrated bicultural identities. Consistent with this perspective, previous research conducted in the United States has linked supportive family relationships to more integrated bicultural identity trajectories among Latinx adolescents (Schwartz et al., 2015). Accordingly, we expected that positive family relationships would facilitate adolescents' engagement in more resolved BII trajectories.

Contrary to this expectation, higher family relationship quality as a main effect was associated with membership in the Compartmentalized BI subgroup (characterized by relatively high levels of perceived Harmony and Compartmentalization) in our sample. Although this finding may initially seem unexpected, it can be understood in light of the German context. In assimilation‐oriented contexts such as Germany (Zick & Krott, 2021), adolescents' endorsement of and identification with the minority culture – which may be reinforced through close family relationships—may contradict societal expectations to assimilate in public domains (i.e., schools, classrooms, teachers). Hence, adolescents are required to navigate these differing cultural expectations and adjust as they move between (cultural) contexts (Arends‐Tóth & van de Vijver, 2006; Phelan et al., 1991; Vietze, Schachner, et al., 2020). The Compartmentalized BI subgroup may represent an adaptive resolution of this challenge (Phelan et al., 1991), which is corroborated by the finding that the Compartmentalized BI subgroup reported medium to high levels of psychological adaptation (Duwe et al., 2026). Hence, separating bicultural identities does not necessarily imply conflict or maladjustment among ethnically minoritized adolescents in Germany. Instead, these findings suggest that blended or fully integrated bicultural identities may not be the only adaptive expression of BII, particularly in assimilation‐oriented national contexts, like Germany.

Beyond the family context, our findings indicate that positive *peer relationships* play a significant role in adolescents' BII trajectories. Consistent with socialization theories, peers represent important contexts in which adolescents learn culturally relevant norms, values, and strategies for navigating social worlds (Titzmann et al., 2024; Vygotsky, 1978). In multicultural peer networks in particular, adolescents can encounter and learn about diverse cultural perspectives and strategies for navigating multiple cultural identities, which may offer important guidance on how cultural identities can be integrated and expressed, as well as how such expressions are received within their peer group. Interestingly, peer acceptance was highest in the Conflict Resolution subgroup (characterized by high levels in all four BII dimensions at T1 and the most pronounced decrease in BII Conflict at T2) and even exceeded levels observed in the High Integrated BI subgroup (characterized by high and stable levels of Blendedness and Harmony), which is typically considered the most adaptive form of BII (Amiot et al., 2007; Benet‐Martínez et al., 2021). This finding suggests that peers in our German sample play a crucial role in resolving bicultural identity conflicts, which is consistent with general identity theories that emphasize the role of peers in processes of identity exploration and commitment (Branje et al., 2021; Rageliene, 2016). Extending this perspective to BII, the resolution of bicultural identity conflicts may represent one pathway through which peers facilitate the development of more coherent and committed (cultural) identities.

Last, positive relationships with the *teacher* may be particularly important for the development of adolescents' bicultural identities because teachers in schools provide a central socialization agent from whom ethnically minoritized adolescents learn about norms and values in the public domain. Moreover, teachers in Germany predominantly belong to the majority culture (Paizan et al., 2024) and, hence, also represent normative and structural positions of social power that shape what is considered legitimate, desirable, and socially rewarded in society (Paizan et al., 2024; Sidanius & Pratto, 1999). Consequently, positive relationships with teachers may support adolescents' engagement with the national culture along with communicating messages that both adolescents' heritage and national identities are valued and can coexist, which should enable them to blend their cultural identities (Benet‐Martínez et al., 2021; Ward, 2013). Consistent with these theoretical considerations, a positive relationship with the teacher was found to predict membership in the High Integrated BI subgroup (main effect). This finding suggests that when teachers treat all students in culturally diverse classrooms with respect and fairness, ethnically minoritized adolescents are more likely to develop blended and harmonious bicultural identities. In a similar vein, Schachner et al. (2016) demonstrated that a positive classroom climate characterized by both equality and inclusion, as well as cultural pluralism, is positively associated with adolescents' identification with both ethnic minority and majority cultures, underscoring the role of institutional support in fostering bicultural identity development.

### Interactions of socialization agents and BII

The significant interactions found in our data further demonstrate that family, peers, and teachers can operate synergistically to create a coherent interpersonal environment that fosters adolescents' identity integration, which supports key propositions of Bronfenbrenner (1981) ecological systems theory as well as ecological developmental models of immigrant‐origin youth (Reich & Vandell, 2011; Suárez‐Orozco et al., 2018). These synergistic effects can be explained through Sameroff's (2009) transactional model of development, which emphasizes that developmental outcomes emerge from reciprocal and reinforcing exchanges between individuals and their various social contexts. For example, positive family relationships can foster secure attachment and provide a safe base from which adolescents can explore their cultural identities (Juang et al., 2018). This may enable adolescents to engage more confidently with multicultural peers and teachers, which, in turn, can increase their opportunities to receive support, acceptance, and affirmation of both their ethnic heritage and national cultural identities outside the family context. Thus, supportive relationships across multiple contexts may create developmental cascades in which experiences in one relational context are resources that can reinforce and extend the benefits of relationship experiences made in other contexts (Sameroff, 2009).

The interaction effects found in this study are consistent with this ecological perspective. For example, while supportive family relationships alone did not significantly relate to more resolved BII trajectories, in combination with teacher relationship quality, they were positively associated with membership in the High Integrated BI trajectory subgroup (characterized by high and stable levels of Blendedness and Harmony). One possible explanation for this pattern is that supportive relationships with both families and teachers provide adolescents with the relational security and support needed to learn about, explore, and negotiate both cultural identities across different social environments, so that an integration of cultural identities becomes more likely.

Conversely, the absence of positive family relationships, especially in combination with less supportive relationships with teachers, was associated with membership in the Ambivalent BII trajectory subgroup (characterized by medium‐high levels in all four BII dimensions and a significant increase in Conflict over time). This finding suggests that adolescents may be most challenged in developing resolved bicultural identities when they lack supportive relational foundations across different culturally affiliated contexts. In particular, when adolescents experience only limited relational support across private (family) and public (school) domains, they may have fewer opportunities to develop a coherent understanding of how their cultural identities relate to one another and how they can be meaningfully integrated and expressed within and across the different domains of their daily lives.

The interaction effects involving peer relationships (positive peer × teacher relationships associating with the High Integrated BI subgroup relative to the Ambivalent BII subgroup, and positive peer × family relationships associating with the Conflict Resolution subgroup, relative to the Ambivalent BII subgroup) similarly support the outlined ecological perspectives (Bronfenbrenner, 1981; Sameroff, 2009; Suárez‐Orozco et al., 2018). Specifically, high peer acceptance seemed to amplify the positive associations of both family and teacher relationships with membership in more resolved BII trajectory subgroups. An explanation for these findings is that peers may serve as a bridging function between adolescents' family and school environments. Multicultural peers (as we had in the school context of our study) are embedded in both contexts; they may help adolescents connect experiences across private (family) and public (school) domains by providing social support, modeling strategies for navigating multiple cultural expectations, and validating experiences across contexts. This interpretation is consistent with research showing that peers can strengthen the influence of parents and teachers on adolescent development (Li et al., 2025; Verhoeven et al., 2019).

Finally, the exploratory three‐way interaction is also broadly consistent with the outlined ecological developmental theories (Bronfenbrenner, 1981; Sameroff, 2009; Suárez‐Orozco et al., 2018). It predicted membership in the High Integrated BI subgroup relative to the Conflict Resolution subgroup. Although both subgroups reflect comparatively resolved forms of BII, this finding suggests that they could represent distinct stages in the BII resolution process: whereas the Conflict Resolution subgroup appears to be in a transitional phase primarily associated with positive peer relations, the High Integrated BI subgroup seems to particularly benefit from synergistic effects of supportive relationships across multiple social contexts, including family, peers, and teachers. Although this finding requires replication, it provides preliminary evidence that coherent and mutually reinforcing social environments across adolescents' primary socialization contexts may facilitate the development of harmonious and blended bicultural identities.

### Practical implications

The practical implication of our research is apparent. Interventions aimed at supporting bicultural identities among ethnically minoritized youth may not need to exclusively target intrapersonal processes (e.g., self‐concept clarity, cultural identity exploration), but may also benefit from fostering secure and supportive relationships among key socialization agents across multiple ecological contexts. Interventions that strengthen supportive ties with families, peers, and teachers may create relational conditions that foster the development of more resolved forms of BII and could support adolescents in overcoming BII‐related ambivalence. Importantly, the relevance of coordinated support across multiple social contexts may extend beyond BII and reflect a more general principle of identity development. For example, some findings from the Identity Project, a school‐based intervention designed to promote adolescents' ethnic‐racial identity development, suggest that effects of identity‐focused interventions may be strengthened when multiple socialization agents are involved. Specifically, families may reinforce school intervention experiences by engaging in ethnic‐racial socialization practices at home (Sladek et al., 2021). Thus, interventions targeting BII and (ethnic‐cultural) identity development more generally may benefit from engaging multiple socialization agents, as changes within one social environment may be strengthened when reinforced across adolescents' broader social ecologies.

### Limitations

This study has several key strengths: The longitudinal, person‐oriented character of the BII trajectory subgroups based on four BII dimensions, the combined view of three core social relations known to be decisive for adolescents' identity (albeit often seen in isolation), and the linking of general relationship quality with (acculturation‐related) processes of identity development (BII). Our findings also enhance the existing understanding regarding the role of social relationships in BII processes during adolescence. Nevertheless, the study is not without limitations. First, the contextual nature of our findings may limit their generalizability. Identity development is inherently embedded within specific social, political, cultural, and historical contexts which influence how identities are formed, experienced, and negotiated (Galliher et al., 2017; Suárez‐Orozco et al., 2018). The present study was conducted in Germany, a national setting characterized by comparatively strong assimilation expectations (Zick & Krott, 2021), which may intensify perceived discrepancies between cultural expectations in private and public domains (Vietze, Schachner, et al., 2020). Accordingly, the observed associations between positive relationships and adolescents' BII may differ in more multicultural or pluralistic societies, where integration is more institutionally supported. Replication across diverse sociopolitical contexts is therefore necessary to better understand the contextual boundaries of these effects.

Second, we did not directly assess the cultural affiliations of specific socialization agents (e.g., family, peers, or teachers), but instead relied on affiliation tendencies across social settings, as suggested by prior research (Arends‐Tóth & van de Vijver, 2006; Hughes et al., 2006; Phelan et al., 1991). Future research would benefit from directly assessing the cultural affiliations and acculturation orientations of these socialization agents and, ideally, employing dyadic or triadic designs to better capture how relational and cultural dynamics unfold within these relationships (Paizan et al., 2026). Such approaches would allow for a more precise understanding of how the cultural affiliations of close others shape adolescents' BII.

Third, while the overall sample size (*N* = 226) was acceptable, subgroup sizes were partly modest, given the existence of five distinct BII subgroups. Although the observed pattern of findings is theoretically meaningful, limited statistical power and small cell sizes may increase the risk of unstable parameter estimates, especially in complex interaction models, such as the three‐way interaction. Hence, results, particularly of the three‐way interaction, should be interpreted with caution, and replications in larger samples with more participants are needed.

Finally, this study examines ethnically minoritized adolescents' bicultural identity development primarily from a majority‐group researchers' perspective. Although the authors aimed to remain sensitive to experiences and structural dynamics that they cannot fully grasp themselves, and engaged in scholarly exchange to incorporate feedback from minoritized researchers in the field, the interpretations of the results may still reflect majority‐positioned assumptions. Future research would benefit from more systematically integrating minoritized perspectives and employing participatory approaches to ensure culturally grounded interpretations of bicultural identity processes.

## CONCLUSION

Building high‐quality relationships represents a normative developmental task that applies to all adolescents. For minoritized youth, however, this task may involve navigating intergroup dynamics to a greater extent, as their relationships often span multiple culturally affiliated contexts. In this sense, supportive relationships with family, peers, and teachers serve not only as general developmental assets for identity formation, but also as a specific resource supporting acculturation processes, such as BII. Drawing on longitudinally derived BII trajectory subgroups based on four BII dimensions and integrating three core social relationships that are typically examined in isolation, our study demonstrates that both the quality of relationships and their interplay across proximal contexts contribute to adolescents' differential BII experiences. Positive peer relationships seem particularly supportive for resolving BII Conflict over time, and simultaneous positive relationships with family, peers, and teachers are associated with more blended and integrated forms of BII.

Ultimately, our findings suggest that integrated bicultural identities may be facilitated when minoritized adolescents experience that both their cultural identities are accepted, supported, and can be meaningfully expressed across and within culturally diverse social contexts in which their lives take place. Thus, fostering supportive relational environments that recognize and provide space for the expression of multiple cultural identities across adolescents' key developmental contexts (including families, *schools, and* peers) may represent an important avenue for supporting adolescents' bicultural identity development.

## AUTHOR CONTRIBUTIONS

**Paula Duwe:** Writing – original draft; methodology; visualization; writing – review and editing; software; formal analysis; project administration; data curation; conceptualization; validation. **Peter F. Titzmann:** Funding acquisition; investigation; writing – review and editing; supervision; resources.

## FUNDING INFORMATION

This research was supported by the Ministry for Science and Culture in Lower Saxony, Germany [grant number 76202‐17‐9/17].

## CONFLICT OF INTEREST STATEMENT

The authors declare no conflicts of interest.

## ETHICS STATEMENT

All procedures were performed in accordance with the ethical standards of the institution's Ethics Committee (Ethics Committee Leibniz University Hannover; EV LUH 04/2019; 03/05/2019).

## CONSENT STATEMENT

Informed consent was obtained from all participants, and their parents were included in the study.

## Supporting information


**Table S1.** Information country groupings in the sample description.
**Table S2**. Descriptive statistics of mean‐centered predictor.
**Table S3**. Plots of significant interactions.
**Table S4**. Multinomial logistic regression with “High Integrated BI” as reference group. Demographic variables included as control variables.
**Table S5**. Multinomial logistic regression with “Ambivalent BII” as reference group. Demographic variables included as control variables.
**Table S6**. Multinomial logistic regression analysis with three‐way interaction included.

## Data Availability

The data that support the findings of this study are available from the corresponding author upon reasonable request.
