# Supplementary material for: How relationship‐quality with family, peers, and teachers relates to adolescents' bicultural identity integration (BII)
Source: J Res Adolesc. 2026 Nov 13;36(4):e70321. doi: 10.1111/jora.70321 (PMC13768793; doi:10.1111/jora.70321)
Supplement: Supplementary file 1 [file JORA-36-0-s001.docx]

**Supplementary Material S1 – S6 for the Manuscript: How Relationship-Quality with Family, Peers, and Teachers relates to Adolescents’ Bicultural Identity Integration (BII)**

**Supplementary Material S1. Table.** Information Country Groupings in the Sample Description.

| **Geographic Region** | **Countries/areas** | **n %** |
| --- | --- | --- |
| Middle East and North Africa (MENA) | Iran (3), Iraq (9), Syria (14), Tunisia (2), Lebanon (5), Morocco (2), Türkiye (43), Georgia (3), Somalia (1) | 82 36.28% |
| Southern Asia (M49) | Afghanistan (11), Pakistan (2), India (1), Sri Lanka (1) | 15 6.64% |
| Central Asia (M49) | Kazakhstan (7) | 7 3.10% |
| Southern Europe (M49) | Serbia (6), Kosovo (5), Bulgaria (4), Bosnia and Herzegovina (1), Romania (5), Albania (2), Montenegro (2), Greece (5) | 30 13.27% |
| Eastern Europe (M49) | Moldova (1), Ukraine (3), Russia (11), Poland (13) | 28 12.39% |
| Northern Europe (M49) | Lithuania (2) | 2 0.88% |
| Western Europe (M49) | France (1), Germany (24) | 25 11.06% |
| Sub-Saharan Africa | Ghana (10), Cameroon (2), Angola (1), Ethiopia (1), Togo (3), Nigeria (3), Congo (1), Senegal (2) | 23 10.18% |
| South-eastern Asia (M49) | Vietnam (1), Cambodia (1) | 2 0.88% |
| South America (M49) | Venezuela (1), Colombia (1), Chile (1), Brazil (1) | 4 1.77% |
| Northern America (M49) | United States (2) | 2 0.88% |
| Not classifiable using M49 | Kurdistan (3) | 3 1.33% |
| Missing |  | 3 1.33% |
| Total |  | 226 100% |

**Supplementary Material S2. Table.** Descriptive Statistics of Mean-Centered Predictor Variables by BII Subgroup Membership.

|  | **Family Climate** | | **Peer Acceptance** | | **Teacher-Generated Classroom-Climate** | |
| --- | --- | --- | --- | --- | --- | --- |
|  | *M* | *SD* | *M* | *SD* | *M* | *SD* |
| **BII Subgroup** |  |  |  |  |  |  |
| High Integrated BI | 0.047 | 1.19 | 5.43^a^ | 1.38 | 5.58^a^ | 0.98 |
| Conflict Resolution | -0.064 | 1.25 | 0.717 | 1.13 | 0.056 | 1.01 |
| Ambivalent BII | -0.195 | 1.25 | -0.133 | 1.42 | -0.343 | 1.03 |
| Compartmentalized BI | 0.202 | 0.90 | -0.294 | 1.47 | -0.007 | 1.10 |
| Low BII Engagement | 0.007 | 1.17 | 0.210 | 1.19 | 0.124 | 0.95 |

**Supplementary Material S3.** Plots of Significant Interactions.

**Supplementary Material S4. Table.** Multinomial Logistic Regression with “High Integrated BI” as Reference Group. Demographic variables included as control variables.

*Note.* OR= Odds Ratio, RG= Reference Group, Immigrant Generation coded as 1 = second generation, 2 = first generation, Gender coded as 1 = female, 2 = male. Gender category 3 (diverse) was coded as missing due to the small number of participants (*n* = 2).

|  | **Ambivalent BII** | | | | | | | **Conflict Resolution** | | | | | **Compartmentalized BI** | | | | | **Low BII Engagement** | | | | |  |
| --- | --- | --- | --- | --- | --- | --- | --- | --- | --- | --- | --- | --- | --- | --- | --- | --- | --- | --- | --- | --- | --- | --- | --- |
|  | | *B* | Wald | *OR* | *p* |  | *B* | | Wald | *OR* | *p* |  | *B* | Wald | *OR* | *p* |  | | *B* | Wald | *OR* | *p* |  |
| **RG: High Integrated BI** | |  |  |  |  |  |  | |  |  |  |  |  |  |  |  |  | |  |  |  |  |  |
| Family Climate | | -.04 | .04 | .95 | .840 |  | -.39 | | 1.19 | .67 | .274 |  | .55 | 4.03 | 1.73 | .045 |  | | -.11 | .16 | .89 | .683 |  |
| Peer Acceptance | | -.13 | .49 | .87 | .482 |  | .79 | | 3.44 | 2.21 | .063 |  | -.30 | 2.30 | .73 | .129 |  | | .34 | 1.68 | 1.41 | .194 |  |
| Teacher Classroom Climate | | -.81 | 9.59 | .44 | .002 |  | -.51 | | 1.44 | .59 | .229 |  | -.46 | 2.96 | .63 | .085 |  | | .01 | .00 | 1.01 | .955 |  |
| Interaction Family x Peer | | -.16 | .96 | .84 | .326 |  | .45 | | 3.47 | 1.57 | .062 |  | .28 | 2.93 | 1.11 | .086 |  | | .22 | 1.57 | 1.25 | .210 |  |
| Interaction Family x Teacher | | .07 | .10 | 1.07 | .748 |  | -.24 | | .61 | .78 | .434 |  | -.37 | 2.02 | .68 | .155 |  | | .42 | 2.33 | 1.53 | .126 |  |
| Interaction Peer x Teacher | | -.25 | 1.20 | .79 | .273 |  | .07 | | .04 | 1.07 | .833 |  | .15 | .58 | 1.16 | .446 |  | | -.58 | 4.28 | .56 | .038 |  |
| Age | | -.17 | .68 | .84 | .409 |  | -.62 | | 3.88 | .53 | .049 |  | -.68 | 9.91 | .50 | .002 |  | | -.20 | .79 | .81 | .372 |  |
| Generation Immigration | | -1.41 | 6.14 | .24 | .013 |  | -.37 | | .20 | .69 | .649 |  | -.31 | .27 | .73 | .599 |  | | 2.11 | 3.47 | 8.29 | .062 |  |
| Gender | | -.48 | 1.05 | .61 | .305 |  | -.05 | | .00 | .95 | .943 |  | .55 | 1.27 | 1.74 | .260 |  | | -.63 | 1.51 | .53 | .218 |  |
| Language Proficiency | | .02 | .00 | 1.02 | .964 |  | -.65 | | 1.08 | .51 | .298 |  | -.16 | .10 | .84 | .750 |  | | -.60 | 1.15 | .54 | .284 |  |

**Supplementary Material S5. Table.** Multinomial Logistic Regression with “Ambivalent BII” as Reference Group. Demographic variables included as control variables.

*Note.* OR= Odds Ratio, RG= Reference Group, Generation Immigration coded as 1 = second generation, 2 = first generation, Gender coded as 1 = female, 2 = male. Gender category 3 (diverse) was coded as missing due to the small number of participants (*n* = 2).

|  | **High Integrated BI** | | | | | | | **Conflict Resolution** | | | | | **Compartmentalized BI** | | | | | **Low BII Engagement** | | | | |  |
| --- | --- | --- | --- | --- | --- | --- | --- | --- | --- | --- | --- | --- | --- | --- | --- | --- | --- | --- | --- | --- | --- | --- | --- |
|  | | *B* | Wald | *OR* | *p* |  | *B* | | Wald | *OR* | *p* |  | *B* | Wald | *OR* | *p* |  | | *B* | Wald | *OR* | *p* |  |
| **RG: Ambivalent BII** | |  |  |  |  |  |  | |  |  |  |  |  |  |  |  |  | |  |  |  |  |  |
| Family Climate | | .04 | .04 | 1.04 | .840 |  | -.34 | | .98 | .70 | .322 |  | .598 | 5.23 | 1.81 | .022 |  | | -.06 | .06 | .93 | .801 |  |
| Peer Acceptance | | .13 | .49 | 1.14 | .482 |  | .92 | | 4.94 | 2.52 | .026 |  | -.17 | .93 | .84 | .333 |  | | .48 | 3.57 | 1.61 | .059 |  |
| Teacher Classroom Climate | | .81 | 9.59 | 2.25 | .002 |  | .29 | | .53 | 1.34 | .467 |  | .35 | 2.10 | 1.41 | .147 |  | | .82 | 7.39 | 2.29 | .007 |  |
| Interaction Family x Peer | | .16 | .96 | 1.18 | .326 |  | .62 | | 6.48 | 1.86 | .011 |  | .45 | 7.15 | 1.57 | .007 |  | | .39 | 4.59 | 1.48 | .032 |  |
| Interaction Family x Teacher | | -.07 | .10 | .92 | .748 |  | -.32 | | 1.10 | .72 | .294 |  | -.45 | 3.34 | .63 | .067 |  | | .35 | 2.08 | 1.42 | .149 |  |
| Interaction Peer x Teacher | | .22 | 1.20 | 1.25 | .273 |  | .30 | | .72 | 1.35 | .394 |  | .38 | 4.31 | 1.46 | .038 |  | | -.35 | 1.73 | .70 | .187 |  |
| Age | | .17 | .68 | 1.18 | .409 |  | -.45 | | 2.10 | .63 | .147 |  | -.51 | 6.28 | .60 | .012 |  | | -.03 | .02 | .96 | .874 |  |
| Generation Immigration | | 1.41 | 6.14 | 4.12 | .013 |  | 1.04 | | 1.81 | .50 | .178 |  | 1.10 | 4.22 | 3.01 | .040 |  | | 3.53 | 9.99 | 34.23 | .002 |  |
| Gender | | .48 | 1.05 | 1.62 | .305 |  | .43 | | .38 | 2.85 | .535 |  | 1.04 | 4.84 | 2.82 | .028 |  | | -.15 | .08 | .85 | .772 |  |
| Language Proficiency | | -.02 | .00 | .97 | .964 |  | -.68 | | 1.23 | 1.54 | .266 |  | -.18 | .14 | .82 | .70 |  | | -.63 | 1.26 | .53 | .262 |  |

**Supplementary Material S6. Table.** Multinomial Logistic Regression Analysis with Three-Way Interaction included**.**

*Note.* OR= Odds Ratio, RG= Reference Group

|  | **High Integrated BI** | | | | | **Conflict Resolution** | | | | | | **Compartmentalized BI** | | | | **Low BII Engagement** | | | | | |  |
| --- | --- | --- | --- | --- | --- | --- | --- | --- | --- | --- | --- | --- | --- | --- | --- | --- | --- | --- | --- | --- | --- | --- |
|  | | *B* | Wald | *OR* (95% *CI*) | *p* |  | *B* | Wald | *OR* (95% *CI*) | *p* |  | *B* | Wald | *OR* (95% *CI*) | *p* | |  | *B* | Wald | *OR* (95% *CI*) | *p* |  |
| **RG: Ambivalent BII** | |  |  |  |  |  |  |  |  |  |  |  |  |  |  | |  |  |  |  |  |  |
| Family Climate | | .00 | .00 | 1.00 (.65-1.54) | .986 |  | -.22 | .39 | .80 (.40-1.60) | .530 |  | .50 | 3.90 | 1.65 (1.00-2.73) | .048 | |  | -.15 | .41 | .85 (.53-1.37) | .519 |  |
| Peer Acceptance | | .04 | .05 | .1.04(.73-1.48) | .820 |  | .84 | 4.70 | 2.32 (1.08-4.97) | .030 |  | -.24 | 2.04 | .78 (.56-1.09) | .153 | |  | .47 | 3.58 | 1.60 (.98-2.60) | .058 |  |
| Teacher Classroom Climate | | .74 | 7.91 | 2.09(1.25-3.51) | .005 |  | 1.06 | 4.23 | 2.89 (1.05-7.98) | .040 |  | .24 | .78 | 1.27 (.74-2.16) | .376 | |  | .80 | 6.10 | 2.27 (1.18-4.20) | .014 |  |
| Interaction Family x Peer | | .15 | .81 | 1.16 (.83-1.61) | .366 |  | .53 | 3.89 | 1.70 (1.00-2.88) | .048 |  | .34 | 4.19 | 1.40 (1.01-1.94) | .041 | |  | .31 | 3.13 | 1.36 (.96-1.92) | .077 |  |
| Interaction Family x Teacher | | -.16 | .54 | .85 (.55-1.31) | .462 |  | -.17 | .47 | .84 (.51-1.38) | .492 |  | -.35 | 1.72 | .70 (.41-1.19) | .189 | |  | .28 | 1.83 | 1.33 (.88-2.01) | .175 |  |
| Interaction Peer x Teacher | | .38 | 4.41 | 1.47(1.02-2.11) | .036 |  | -.14 | .19 | .86 (.45-1.65) | .661 |  | .33 | 3.60 | 1.40 (.98-1.99) | .058 | |  | -.37 | 2.24 | .69 (.42-1.12) | .134 |  |
| Int Family x Peer x Teacher | | .07 | .24 | 1.07 (.80-1.44) | .619 |  | -.37 | 3.06 | .68(.45-1.04) | .080 |  | .226 | 2.01 | 1.25 (.91-1.71) | .156 | |  | .02 | .16 | 1.02 (.74-1.40) | .898 |  |
|  | **Ambivalent BII** | | | | | **Conflict Resolution** | | | | | | **Compartmentalized BI** | | | | **Low BII Engagement** | | | | | |  |
|  | | *B* | Wald | *OR* (95% *CI*) | *p* |  | *B* | Wald | *OR* (95% *CI*) | *p* |  | *B* | Wald | *OR* (95% *CI*) | *p* | |  | *B* | Wald | *OR* (95% *CI*) | *p* |  |
| **RG: High Integrated BI** | |  |  |  |  |  |  |  |  |  |  |  |  |  |  | |  |  |  |  |  |  |
| Family Climate | | -.00 | .00 | .99 (.64-1.53) | .986 |  | -.22 | .38 | .79 (.38-1.63) | .537 |  | .50 | 3.38 | 1.65 (.96-2.81) | .066 | |  | -.15 | .36 | .85 (.51-1.42) | .544 |  |
| Peer Acceptance | | -.04 | .05 | .95 (.67-1.37) | .820 |  | .80 | 4.07 | 2.22 (1.02-4.85) | .043 |  | -.28 | 2.33 | .75 (.52-1.08) | .127 | |  | .43 | 2.62 | 1.53 (.91-2.58) | .105 |  |
| Teacher Classroom Climate | | -.74 | 7.91 | .47 (.28-.79) | .005 |  | .32 | .38 | 1.38 (.50-3.82) | .533 |  | -.50 | 2.94 | .60 (.34-1.07) | .086 | |  | .06 | .03 | 1.06 (.53-2.09) | .861 |  |
| Interaction Family x Peer | | -.15 | .81 | .86 (.62-1.19) | .366 |  | .38 | 1.92 | 1.46 (.85-2.50) | .166 |  | 19 | 1.24 | 1.20 (.86-1.68) | .264 | |  | .15 | .74 | 1.17 (.81-1.68) | .389 |  |
| Interaction Family x Teacher | | .16 | .54 | 1.17 (.76-1.81) | .462 |  | -.01 | .00 | .98 (.57-1.69) | .966 |  | -.19 | .46 | .82 (.47-1.42) | .493 | |  | .44 | 3.23 | 1.56 (.96-2.55) | .072 |  |
| Interaction Peer x Teacher | | -.38 | 4.41 | .67(.47-.97) | .036 |  | -.53 | 2.40 | .58 (.29-1.15) | .121 |  | -.05 | .06 | .95 (.65-1.38) | .793 | |  | -.75 | 8.27 | .46 (.27-.78) | .004 |  |
| Int Family x Peer x Teacher | | -.07 | .24 | .92 (.69-1.24) | .619 |  | -.44 | 4.09 | .64 (.41-.98) | .043 |  | .15 | .83 | 1.16 (.84-1.60) | .361 | |  | -.05 | .09 | .94 (.66-1.35) | .764 |  |
